# Automated segmentation of the substantia nigra, subthalamic nucleus and red nucleus in 7 T data at young and old age

**DOI:** 10.1016/j.neuroimage.2016.06.039

**Published:** 2016-10-01

**Authors:** Eelke Visser, Max C. Keuken, Birte U. Forstmann, Mark Jenkinson

**Affiliations:** a FMRIB Centre, Nuffield Department of Clinical Neurosciences, University of Oxford, Oxford, United Kingdom; b Amsterdam Brain and Cognition, University of Amsterdam, Amsterdam, Netherlands

## Abstract

With recent developments in MR acquisition at 7 T, smaller brainstem structures such as the red nuclei, substantia nigra and subthalamic nuclei can be imaged with good contrast and resolution. These structures have important roles both in the study of the healthy brain and in diseases such as Parkinson's disease, but few methods have been described to automatically segment them. In this paper, we extend a method that we have previously proposed for segmentation of the striatum and globus pallidus to segment these deeper and smaller structures. We modify the method to allow more direct control over segmentation smoothness by using a Markov random field prior. We investigate segmentation performance in three age groups and show that the method produces consistent results that correspond well with manual segmentations. We perform a vertex-based analysis to identify changes with age in the shape of the structures and present results suggesting that the method may be at least as effective as manual delineation in capturing differences between subjects.

## Highlights

• We extend MIST by introducing a more flexible MRF-based shape model.
• We segment the substantia nigra, subthalamic nucleus and red nucleus in 7 T data.
• The method produces high-quality results in different age groups.
• We investigate how image intensity influences manual and automatic segmentation.

## Introduction

With recent developments in high-resolution imaging it has become possible to image smaller brainstem nuclei, such as the red nuclei, the substantia nigra and the subthalamic nuclei. In addition to their small sizes, these structures are characterised by their relatively high iron content (Hallgren and Sourander, 1958, Sofic et al., 1991). They exhibit virtually no contrast on *T*_1_-weighted scans, but due to the presence of iron, they are clearly visible on *T*_2_-weighted scans (Drayer et al., 1986, Dormont et al., 2004, Lenglet et al., 2012). Because of the iron, the magnetic susceptibility of these structures is substantially different from that of the surrounding tissue and this means that they are clearly visible in the phase component of gradient echo images (Manova et al., 2009). Phase-based techniques such as susceptibility-weighted imaging (SWI) and quantitative susceptibility mapping (QSM) work especially well at 7 T and can produce very high-resolution images of our structures of interest (Wang and Liu, 2015, Du et al., 2015, Haacke et al., 2004, Forstmann et al., 2014, Keuken et al., 2014).

The subthalamic nucleus and substantia nigra play important roles in the motor system and are of interest in both research and clinical practice as they are affected in Parkinson's disease (PD) (Castrioto et al., 2014, Temel et al., 2006). The subthamalic nucleus is of significant interest clinically as it is a frequently used target for deep brain stimulation (Limousin et al., 1995, Limousin et al., 1998, Kumar et al., 1998, Moro et al., 1999). A large body of literature exists on finding the location of the subthalamic nuclei as this structure is of particular interest in deep-brain stimulation (Zonenshayn et al., 2000, Brunenberg et al., 2011). These methods focus on the larger-scale problem of identifying the structure in a scan, rather than providing an accurate fine-scale delineation of the structure. To locate the structure, these methods use either the image contrast of the substantia nigra itself, or the locations of other brain structures that are more readily identifiable on typical clinical scans.

In this paper, we will be concerned with the smaller-scale problem of delineating the subthalamic nucleus, as well as the substantia nigra and the red nucleus. The amount of published work about automatic segmentation of these structures is limited. This is likely, at least in part, to be a result of the fact that high-quality imaging of the type described by Lenglet et al. (2012); Forstmann et al. (2014) is a fairly recent development. Nevertheless, automatic segmentation can offer important advantages over manual delineation. It dramatically reduces the amount of manual work required and can eliminate operator bias in segmenting the images. Both of these aspects are particularly relevant in larger studies, where the amount of work needed to create manual delineations can be very substantial and where this work is therefore likely to be divided among multiple operators.

Xiao et al. (2012) describe a method that combines *T*_1_- and *T*_2_^⁎^-weighted scans into a single image and use this combined contrast to achieve better non-linear registration of a single atlas, from which the final segmentation is derived. Haegelen et al. (2013) compare two different nonlinear registration algorithms that are driven by *T*_1_-weighted data and in addition apply a patch-based segmentation method to *T*_2_-weighted scans. A label fusion approach was described by Xiao et al. (2014) and Li et al. (2016) use a level set method to segment the subthalamic nucleus. The method described by Kim et al. (2014) combines an active surface model with prior shape knowledge based on manual segmentations in a training set and constraints to prevent neighbouring structures from overlapping.

We have previously described a flexible multimodal method for segmenting the putamen, caudate nucleus and globus pallidus (MIST, Visser et al. (2016)) and in this paper we will extend the methodology by introducing a Markov random field and use it to segment the red nucleus, substantia nigra and subthalamic nucleus. The method is well-suited for the present segmentation problem as different contrasts, such as *T*_2_^⁎^-weighted and phase-derived images, can be used simultaneously by the method. An additional advantage of the method is that it only requires a single reference segmentation from which an initial mesh is constructed. The remainder of the training process uses a set of rule-based priors to automatically detect the boundaries of a structure in the training data. Because of these properties, it is straightforward to retrain the method on different populations and with different image contrasts. This is especially relevant in the presence of pathology, where a model trained on scans of healthy participants may not be representative of the study population. It also means that the method is not strongly tied to the acquisition protocols and data processing used in this paper.

To evaluate the method, we will use high resolution *T*_2_^⁎^-weighted FLASH data on which quantitative susceptibility mapping (QSM) has been performed (Forstmann et al., 2014, Keuken et al., 2014). The *T*_2_^⁎^-weighted magnitude images at different echo times and the QSM image can be used with our method as multiple modalities, taking advantage of the fully multimodal nature of the method. The dataset contains scans from participants of very different ages, and this will allow us to evaluate the method in the presence of atrophy and age-related changes in image intensity.

## Methods

We will segment the red nucleus, substantia nigra and subthalamic nucleus in high-resolution 7 T data using a new extension of a segmentation method that we have described previously and which we have used to segment the striatum and globus pallidus (Visser et al., 2016). The dataset, a subset of which was used in the cited paper, is freely available and has been described in detail by Forstmann et al. (2014). We refer to this paper for a detailed discussion of the data and only reproduce the characteristics that are relevant for the present work.

### Participants

The dataset consists of scans for 53 healthy participants in three age groups, the details of which are given in Table 1. This is in contrast to Visser et al. (2016), in which only the young subjects were used.

### Data acquisition

The data were acquired on a 7 T Siemens Magnetom system (Siemens Healthcare, Erlangen, Germany) with a 24-channel Nova head coil (NOVA Medical Inc., Wilmington MA) (Forstmann et al., 2014). We will mainly be concerned with the *T*_2_^⁎^-weighted multi-echo 3D FLASH component of the acquisition protocol, as this produced the images that we will perform segmentation on. A 128-slice slab, tilted at −23^∘^, was acquired with 0.5 mm isotropic resolution, repetition time (TR) = 41 ms, three echo times (TE1/2/3) = 11.22/20.39/29.57 ms, flip angle = 14^∘^ and bandwidth = 160 Hz/px.

The dataset also includes a whole-brain *T*_1_-weighted MP2RAGE (Marques et al., 2010) scan with 0.7 mm isotropic resolution. In addition, a slab of the same thickness as the FLASH acquisition was acquired using MP2RAGE at 0.6 mm isotropic resolution. The details of these acquisitions, as well as more details regarding the FLASH scan can be found in Forstmann et al. (2014) and Keuken et al. (2014). The QSM was calculated using the phase information of the first echo time FLASH sequence and the method proposed by Schweser et al. (2013). In short the phase data were unwrapped using a Laplacian algorithm, the resulting data were high-pass-filtered using the SHARP approach (Schweser et al., 2011) and finally the filtered phase images were used to calculate the QSM using the Superfast Dipole Inversion (SDI) approach by thresholding the convolution kernel. The threshold was *δ* = 2/3 and is based on previous work by Schweser et al. (2013). The coil combination of the phase data was done automatically by the scanner vendor software (version VE11) and results in some minor phase singularities. These singularities are accounted for by the SHARP algorithm (Schweser et al., 2013).

### Preprocessing and manual segmentation

The whole brain combined MP2RAGE volumes are used to find a nonlinear registration to the 2 mm resolution version of the MNI152 template using FLIRT and FNIRT (Jenkinson and Smith, 2001, Jenkinson et al., 2002, Andersson et al., 2010). The resulting transformation is used later to register MIST's reference mesh to a participant's scans. The MP2RAGE volumes are also registered to the FLASH volumes for each participant and these transformations are combined to obtain a nonlinear transformation from native FLASH to MNI152 coordinates. A cerebrospinal fluid (CSF) mask is created from the MP2RAGE slab using FAST (Zhang et al., 2001).

All three structures have been manually delineated by two different raters. The first rater was the same person for all of the structures, whereas the second set was produced by a different rater for each structure. The second set of manual segmentations of the substantia nigra was produced by different raters for the young group and the two other age groups. The segmentation procedure was described in detail by Keuken et al., 2014, Keuken et al., 2016.

### Segmentation method

**Fig. 1:**
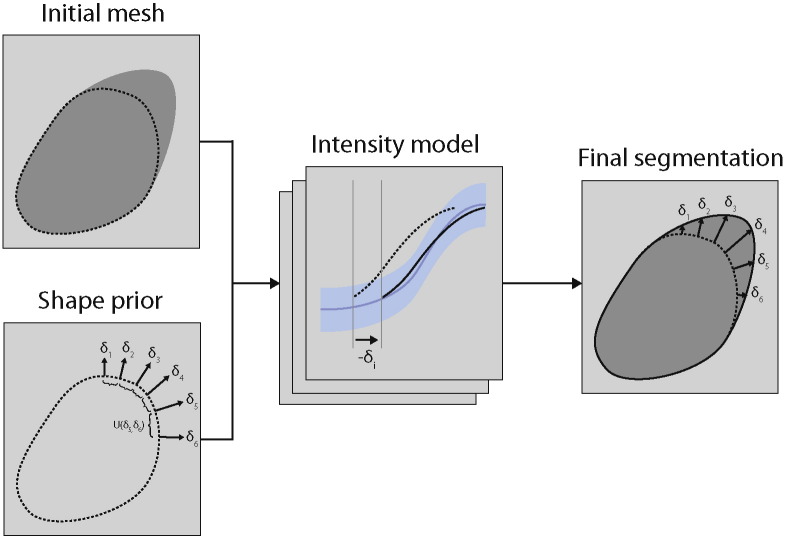
Overview of MIST. The initial mesh (dotted line, top left panel) is only roughly aligned to the anatomical structure (dark gray area). At each vertex of this mesh, a perpendicular intensity profile is measured (dotted profile, middle panel) and aligned to the mean profile learned in the training stage (blue profile, middle panel) to find the displacement that aligns the vertex with the anatomy. The displacements of neighbouring vertices are coupled through the MRF shape prior (lower left panel) and combining the profile likelihood (middle panel) with this prior yields the final segmentation (right panel). Full details of the intensity model are given in Visser et al. (2016). Note that the segmentation method is multimodal and multiple intensity profiles are measured at each vertex; this was omitted from the figure for clarity.

The segmentation method that we will use is based on MIST (Visser et al., 2016). The two main components of this method are an intensity model, which describes the appearance of edges near the boundary of the structure to be segmented and a shape model, which describes deformation of the reference shape (Fig. 1). For the training stage, it only requires a single reference mesh (see below) and a set of unlabelled training volumes, which can be the same images that are to be segmented.

The intensity model is the part of the method that allows it to find the boundaries of a structure in the imaging data. Briefly, our approach measures intensity profiles perpendicular to an initial mesh that is only roughly aligned to an anatomical structure. The Bayesian framework described in Visser et al. (2016) allows us to learn the expected appearance of edges from unlabelled training data given a set of priors that encode our beliefs about these appearances (e.g., magnitude, sign and sharpness of intensity difference across the boundary). Once the appearances of the edges have been learnt, they can be used to find the displacements of the profiles observed at each vertex of a new image, encoded via a likelihood function. These displacements are equal to the local distances between the roughly aligned initial mesh and the estimated anatomical boundary. Information from multiple modalities is automatically weighted within the Bayesian framework, based on the training data (a set of example images, without any manual tracings required) and hence adapts to the specific sequences being used. The rules for setting up the priors automatically will be described below in Section 2.6.

In this paper, we will enhance MIST by replacing the original multivariate normal shape model by a Markov random field (MRF). The multivariate normal model modelled correlations between all pairs of vertices. Here, we have revised the method to make the influence of the shape model more local. This allows for more direct control over the smoothness of the segmentations while allowing the larger-scale shape of the structure to de determined by information in the images.

The new shape model can be considered to act as a multivariate prior on the displacements *δ*_*i*_ at the different vertices, which are otherwise independent (Fig. 1). The displacements are along the local surface normals and by assigning higher probabilities to configurations with similar displacements for neighbouring vertices, the segmentation algorithm can be made to prefer smooth segmentations. To achieve this, we will define an MRF on the triangle mesh of the reference shape. It takes the form

(1) $$p\left(\delta \right)\propto e^{-\sum_{\left(i,j,k\right)\in T}‍U\left(\delta_{i},\delta_{j},\delta_{k}\right)},$$

where *i*, *j* and *k* are the vertex indices that make up a single triangle in the set of triangles *T*, which consists of all the triangles that make up the mesh. The normalising sum over all configurations has been omitted. Eq. (1) is simply the product of the configuration probabilities of all individual triangles. The function *U*(*δ*_*i*_, *δ*_*j*_, *δ*_*k*_) is the negative logarithm of the single-triangle probability and is defined as

(2) $$U\left(\delta_{i},\delta_{j},\delta_{k}\right)=w\left({\left(\delta_{i}-\bar{\delta }\right)}^{2}+{\left(\delta_{j}-\bar{\delta }\right)}^{2}+{\left(\delta_{k}-\bar{\delta }\right)}^{2}\right)$$

with $\bar{\delta }=\frac{\delta_{i}+\delta_{j}+\delta_{k}}{3}$. This is simply the variance of the displacements at the three vertices of the triangle; it is zero if *δ*_*i*_ = *δ*_*j*_ = *δ*_*k*_ and has higher values, which correspond to lower probabilities, for configurations with different values for *δ*_*i*_, *δ*_*j*_ and *δ*_*k*_. The user-specified parameter *w* controls the width of the distribution and allows the specification of the desired level of smoothness (see Table 3).

The training process for the full model is slightly simplified compared to the original procedure, as the new MRF shape model does not require training. Apart from this, both the training stage and the final segmentation stage proceed in the same way as for the original method. During segmentation, an iterated conditional modes algorithm is used to find the maximum a posteriori (MAP) displacements (Besag, 1986). This type of algorithm can only identify local minima, but this does not appear to be an issue in practice due to the fact that the probability mass functions of the displacements *δ*_*i*_, as obtained from the intensity models, are relatively smooth. Furthermore, the displacements are initialised to the maxima obtained from the intensity model without taking the MRF into account, meaning that the optimum should be relatively close to the initial values.

### Mesh generation

MIST requires a single reference mesh as a starting point for each structure that is to be segmented. For the original method, we derived these meshes from the Harvard-Oxford subcortical atlas. This atlas does not include the red nucleus, substantia nigra and subthalamic nucleus however, and because of this we perform one manual segmentation on the group average volume. This average is created by nonlinearly registering the FLASH echo 3 images for all participants to MNI152 space, using the MP2RAGE scan as an intermediate image, and then averaging the registered volumes. The three structures are then manually segmented in both hemispheres on the group average image. The resulting voxel masks are eroded using a 3x3x3 voxels box kernel, as this helps to prevent minor topological problems. They are then converted to meshes using a procedure similar to the one used for the original method. Meshes for the substantia nigra and red nuclei are generated after resampling the manual segmentations to 1 mm^3^ resolution, while the meshes for the subthalamic nuclei are produced from the masks at their original resolution (0.5 mm isotropic) because of the smaller size of these structures. Both resolutions are higher than the 2 mm^3^ resolution used in Visser et al. (2016) to allow the method to accurately segment the smaller structures that are targeted in the current paper. It is worth noting that the reference meshes are not specific to this study or the image contrasts used here and can be used to delineate the same structures in different datasets.

### Parameter setup

The specification of the intensity model priors is performed automatically using the set of rules in Table 2. These rules are used to automatically set up the intensities that the model expects based on the intensity values in the training data inside a number of atlas-derived regions of interest. When applying the method to different datasets, they can be reused without user intervention, as long as the scans have a nominally similar contrast to the volumes used in this paper (i.e. *T*_2_-/*T*_2_^⁎^-weighted and/or QSM). A detailed explanation of the automated setup procedure is given in Visser et al. (2016). The data for all participants are used to automatically set up the prior parameters for the training stage and to train the model.

The covariance priors are set up in a similar automatic fashion according to Table 3, which also lists values for the other parameters of the model. These parameters are set similarly to the values used in the original MIST paper and we refer to that paper for a discussion of their relevance. The parameter *n*_0_ is set to a lower value as there is no apparent advantage to using a higher weighting for the intensity priors. The new parameter *w*, which specifies the weight of the MRF, is set empirically to a level where the smoothing removes jaggedness, but does not substantially affect the overall shape of the segmentation. For the subthalamic nucleus a higher value for *w* is needed to obtain a similar level of smoothness, as a higher resolution mesh was used for this structure.

### Analysis of segmentation performance

To assess the accuracy of the segmentations produced by our method, we will compare the resulting masks with the manual segmentations using the Dice overlap score (Dice, 1945). Masks are generated from the automatic segmentations by including all voxels whose centre is inside the final mesh. The manual masks produced by the two different raters are compared independently to the automatic masks, as well as to each other.

A common reason for using either manual or automatic segmentation in imaging research is to identify correlations between the volume of a structure and some condition, such as disease state. For such applications, an important property of a method is the degree to which it captures the anatomical differences between participants. This is in contrast to the Dice score and to the surface-based distances that are sometimes used, as both of these are very sensitive to consistent differences between automatic and manual segmentation. An example of this would be a method that consistently places the boundary of a structure to the outside of the manual mask by a small distance. Such differences are of little relevance when correlating with disease or another condition, but have a large effect on Dice scores and can easily obscure any differences in performance relating to actual anatomical variability. We will investigate how successful MIST is in capturing anatomical variability by correlating the volumes of the automatic segmentations with the volumes obtained using manual labelling.

Differences in image intensity between participants may potentially confound the volumes reported by both automatic and manual segmentation. This issue is particularly relevant given the wide age range of the participants in the present study and the associated iron-related intensity differences. To investigate how such differences influence the final segmentations, we will investigate the relationship between the normalised modal image intensity inside the segmented structure and the volume of the segmentation. Intensities are measured within the eroded manual intersection masks. For this analysis, the intensities inside the structures are normalised to remove trivial differences in global scaling in the *T*_2_^⁎^-weighted volumes. This is achieved through division by the average white matter signal intensity as computed using the white matter mask produced by FAST (Zhang et al., 2001) from the MP2RAGE slab. The QSM volumes are not normalised as they do not exhibit arbitrary global scaling and we can directly use the intensities inside the structure. In the analyses of volume and image intensity we will use the intersection of the masks produced by the two raters and average over the left and right hemispheres. All plots are generated using R (http://www.r-project.org/).

### Shape analysis

In addition to comparing the volume of structures between age groups, we will also investigate if there are any changes in the shapes of the structures. We will do this by testing for each vertex whether the surface of the structure is displaced inwards or outwards in the young group compared to the two older groups. The analysis will be performed independently in both hemispheres.

Different participants' segmentations of a given structure are based on the same reference mesh and therefore have corresponding vertex indices. Furthermore, the displacements found during the segmentation process will only move vertices along the local normals. This means that even for the final meshes, there is anatomical correspondence between participants of vertices with the same index. We can find the surface displacement by calculating, for each vertex, the distance from the location of that vertex to the reference mesh. This calculation is performed using VTK's vtkImplicitPolyDataDistance filter (http://www.vtk.org/). Prior to the calculation of these distances, each participant's segmentation needs to be registered to the reference mesh. The spherical shape of the red nucleus and the somewhat cylindrical shape of the subthalamic nucleus make it difficult to allow for rotations. Because of this, we will only allow translation in the registration procedure. To remove global anisotropic scaling, we will apply the affine transformations to MNI space before registering and comparing the meshes (Patenaude et al., 2011).

Statistics for the shape analysis are performed using the general linear model (GLM)-based formulation that is common throughout neuroimaging. Permutation analysis of linear models (PALM, Winkler et al. (2014)) is used to perform inference on the model using a 2D mesh-based version of threshold-free cluster enhancement (TFCE, Smith and Nichols (2009)) to correct for multiple comparisons by controlling the familywise error rate (FWER).

## Results

### Overlap with manual segmentation

**Fig. 2:**
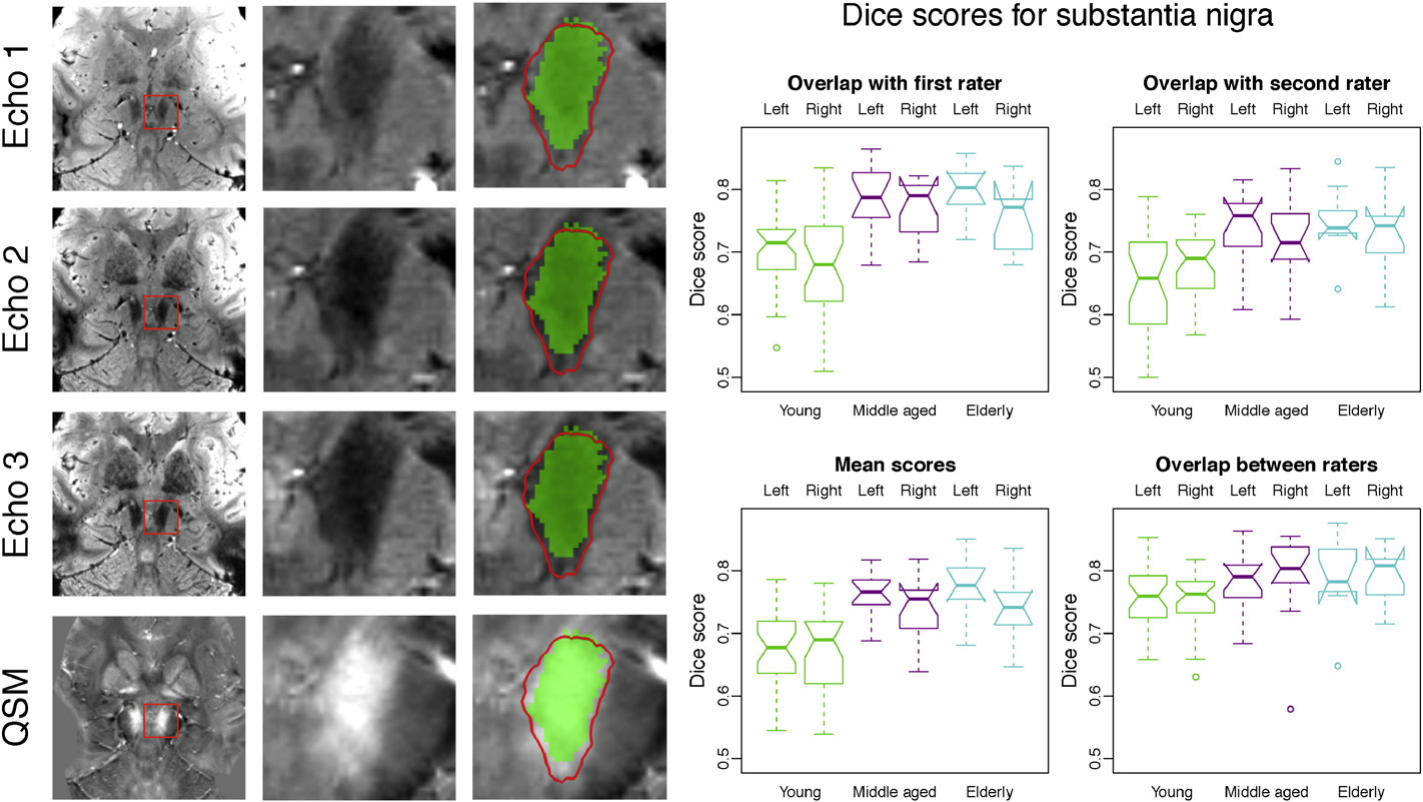
Example segmentation and Dice scores for the substantia nigra. First three columns: Example segmentation in a young participant by rater 1. The automatic segmentation result is shown as a red contour and the manually labelled mask is shown in green. Columns 4 and 5 show Dice scores per age group for rater 1 and 2, the average of these scores and the overlap between the two raters. See the boxplot notches for the statistical significance of differences between age groups and segmentation types (points in the boxplots whose distance to the box is more than 1.5 times the interquartile range are treated as outliers. The notches are constructed in such a way that non-overlapping notches indicate a significant difference in the medians (*p* < 0.05). Note that the ends of the boxes may appear inverted if the notches are wider than the box. Details about the calculation of the notch extents are given in the documentation for the boxplot.stats function in R (http://www.r-project.org/)).

A comparison between automatic and manual segmentation of the substantia nigra is shown in Fig. 2. The slices in the panels on the left show that there is good overlap of the automatically produced mesh with the manually labelled mask. Automatic segmentation tracks the boundaries of the substantia nigra accurately in the FLASH volumes and successfully captures the area of high susceptibility in the QSM reconstruction. Dice scores for the substantia nigra are shown in the panels on the right. The scores for overlap with the masks produced by rater 1 are slightly higher than those for rater 2. It is remarkable that scores in the middle-aged and elderly groups are higher than those in the young group, both for the comparison of automatic to manual segmentations and for the comparison between manual segmentations by different raters. A potential reason for this could be the weaker contrast at younger age as less iron is present. We will come back to this later.

**Fig. 3:**
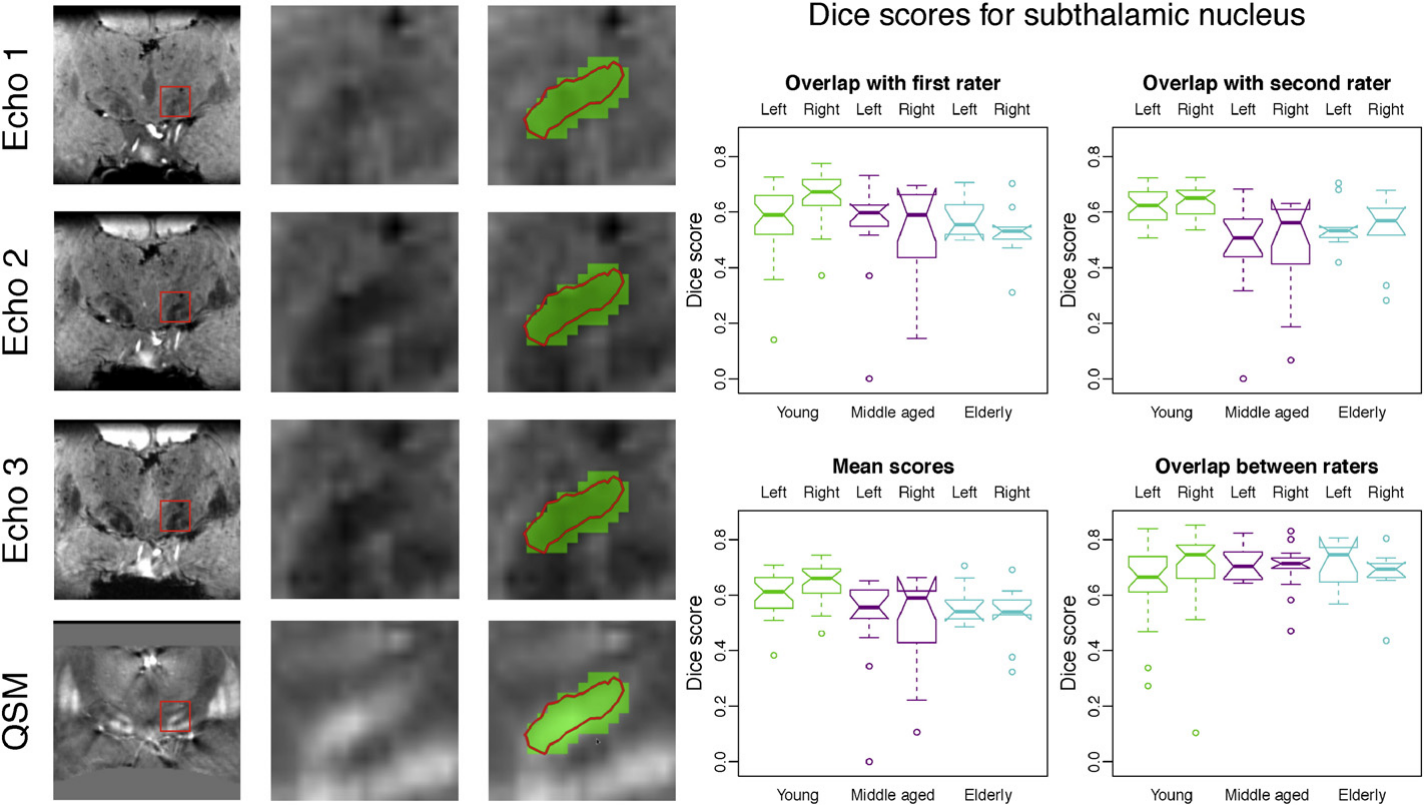
Example segmentation and Dice scores for the subthalamic nucleus. First three columns: Example segmentation in a young participant by rater 1. The automatic segmentation result is shown as a red contour and the manually labelled mask is shown in green. Columns 4 and 5 show Dice scores per age group for rater 1 and 2, the average of these scores and the overlap between the two raters. See the boxplot notches for the statistical significance of differences between age groups and segmentation types (points in the boxplots whose distance to the box is more than 1.5 times the interquartile range are treated as outliers. The notches are constructed in such a way that non-overlapping notches indicate a significant difference in the medians (*p* < 0.05). Note that the ends of the boxes may appear inverted if the notches are wider than the box. Details about the calculation of the notch extents are given in the documentation for the boxplot.stats function in R (http://www.r-project.org/)).

Automatic segmentation of the subthalamic nucleus yields a mesh that is qualitatively similar to manual segmentation, although the manually labelled mask is slightly more inclusive in the example participant (Fig. 3). Dice scores are lower than for the substantia nigra, which is likely to be due to the considerably smaller size of the structure. The overlap scores between the two raters are only slightly higher than the overlap between automatic and manual segmentation, confirming that the Dice scores attained by MIST represent good performance for a structure of this size.

**Fig. 4:**
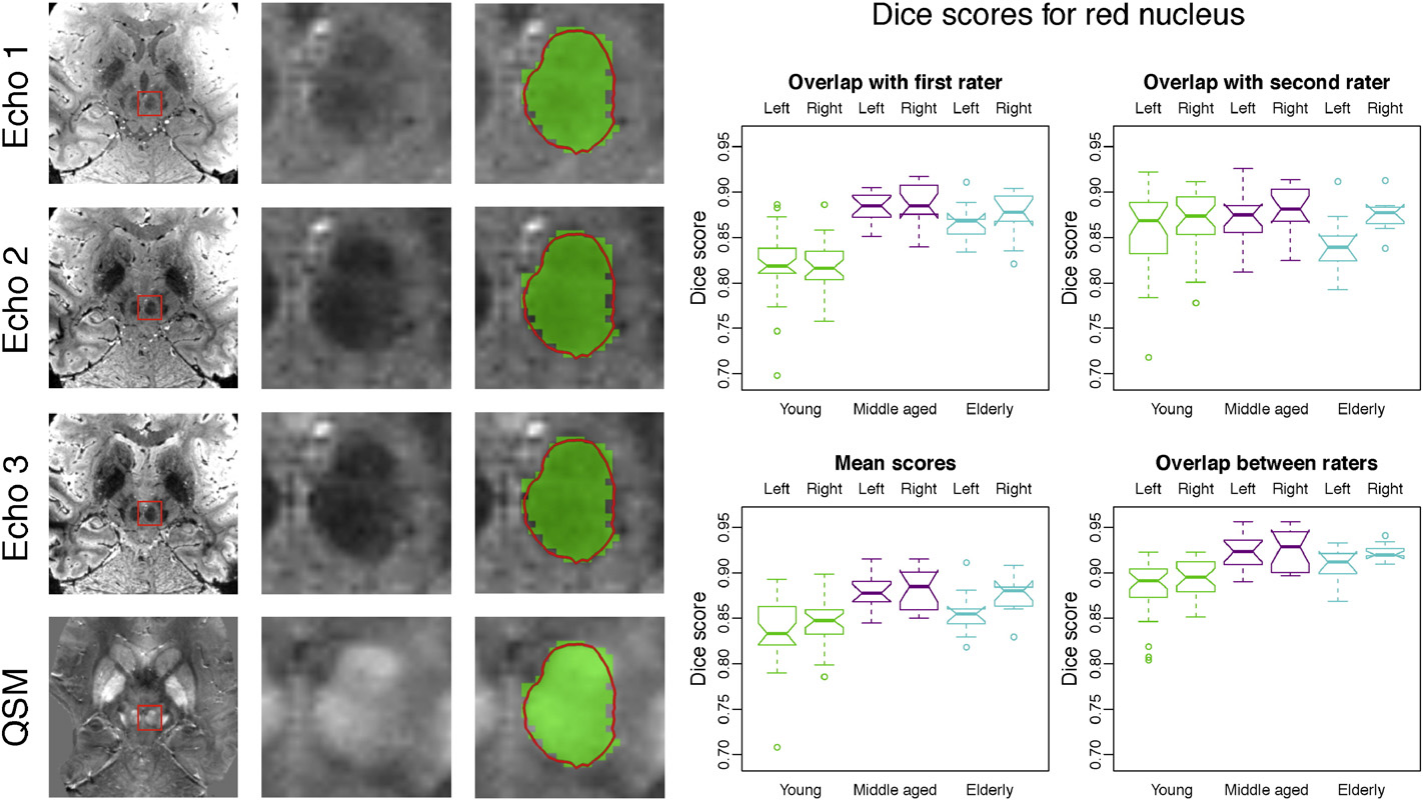
Example segmentation and Dice scores for the red nucleus. First three columns: Example segmentation in a young participant by rater 1. The automatic segmentation result is shown as a red contour and the manually labelled mask is shown in green. Columns 4 and 5 show Dice scores per age group for rater 1 and 2, the average of these scores and the overlap between the two raters. See the boxplot notches for the statistical significance of differences between age groups and segmentation types (points in the boxplots whose distance to the box is more than 1.5 times the interquartile range are treated as outliers. The notches are constructed in such a way that non-overlapping notches indicate a significant difference in the medians (*p* < 0.05). Note that the ends of the boxes may appear inverted if the notches are wider than the box. Details about the calculation of the notch extents are given in the documentation for the boxplot.stats function in R (http://www.r-project.org/)).

For the red nucleus, manual and automatic segmentation correspond very well (Fig. 4). The red nucleus has very well-defined borders, which means there are few ambiguities and as a results of this, there are only small differences between manual and automatic segmentation.

### Comparison of segmented volumes as found using automatic and manual segmentation

**Fig. 5:**
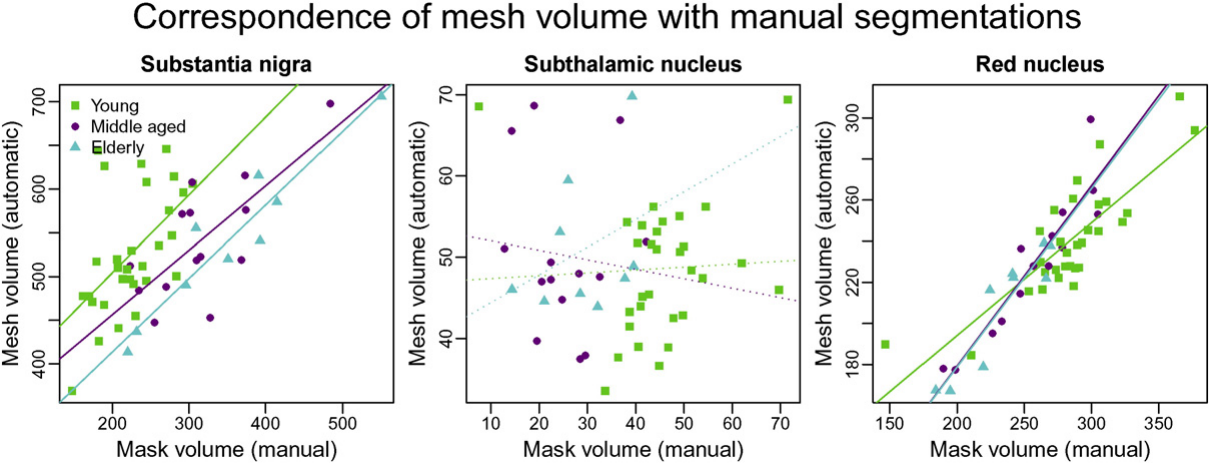
Comparison of substantia nigra, subthalamic nucleus and red nucleus volumes as determined using manual and automatic segmentation. Each point represents a participant, coloured by age group (green: young, magenta: middle-aged, cyan: elderly). Regression lines are solid if significant (*p* ≤ 0.05) and dotted otherwise.

A comparison between the volumes of the substantia nigra and red nucleus as determined using manual and automatic segmentation shows that for these structures, both methods correspond to a large degree (Fig. 5). This indicates that automatic segmentation successfully captures the anatomical variability that underlies the volume differences in the manually labelled masks.

The volumes of the manual and automatic segmentations of the subthalamic nucleus do not exhibit significant correlations. This indicates that the variability present in either the automatic or manual segmentations (or both) does not represent anatomical variations. The difference between the smallest and largest manual segmentations is remarkably large, which may reflect difficulty in labelling the structure in a consistent fashion. This interpretation is also supported by the relatively large differences between raters and between age groups for the manual segmentations in Fig. 10 below. The uncertainty in the exact borders of the structures is likely to arise from limitations in contrast-to-noise ratio and resolution. Note that consistent global under- or over-estimation cannot explain the lack of correlations for the subthalamic nucleus in Fig. 5 as it would change the intercept, but have only a small effect on the slope.

### Shape analysis

**Fig. 6:**
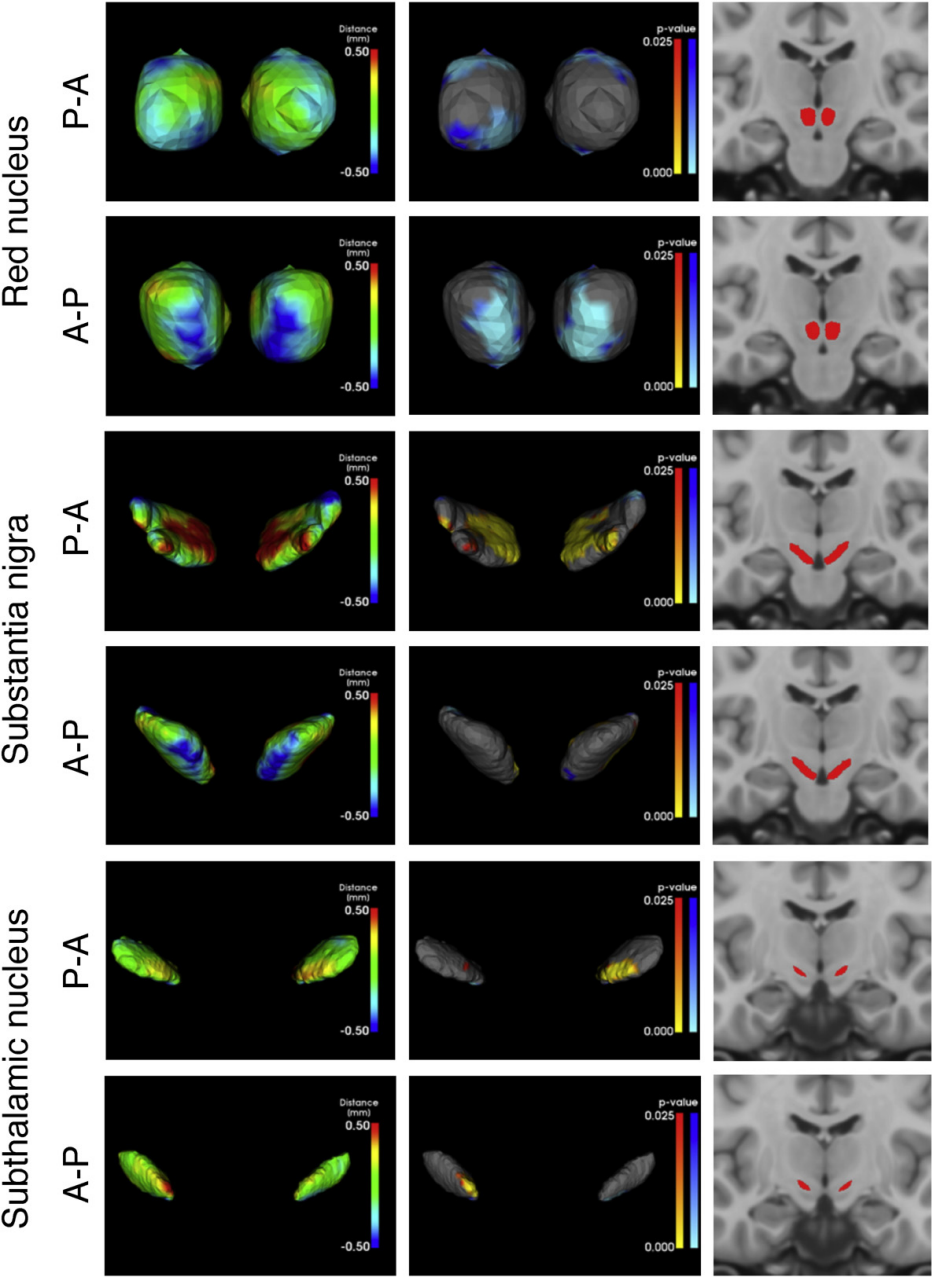
Shape analysis of substantia nigra, subthalamic nucleus and red nucleus. Segmentations were affine-registered to MNI space prior to comparison. The tested contrast is Old > Young, where old includes both the middle-aged and elderly groups. The left column shows the difference in means between the two groups and the right column shows the associated *p*-values. In the right column, red-yellow colours indicate a significant local volume increase with age and blue colours indicate a decrease (*p* ≤ 0.025 for either the positive or negative contrast, corrected using TFCE).

The shape analysis in Fig. 6 shows how the shape of the substantia nigra, subthalamic nucleus and red nucleus differs between the young participants and a combined group containing the middle-aged and elderly participants. This analysis is based on the automatic segmentations produced by MIST. For the red nucleus, there is a band around the structure where it is smaller in the older group, i.e. the surface has moved inwards. This seems to indicate that the structure shrinks in the anterior-posterior and inferior-superior directions, thereby becoming more elongated in shape in the medial-lateral direction. The main change in the substantia nigra is that its thickness appears to increase with age. In the subthalamic nucleus, there appears to be a small enlargement with age on the medial side of the structure.

### Intensity variations

**Fig. 7:**
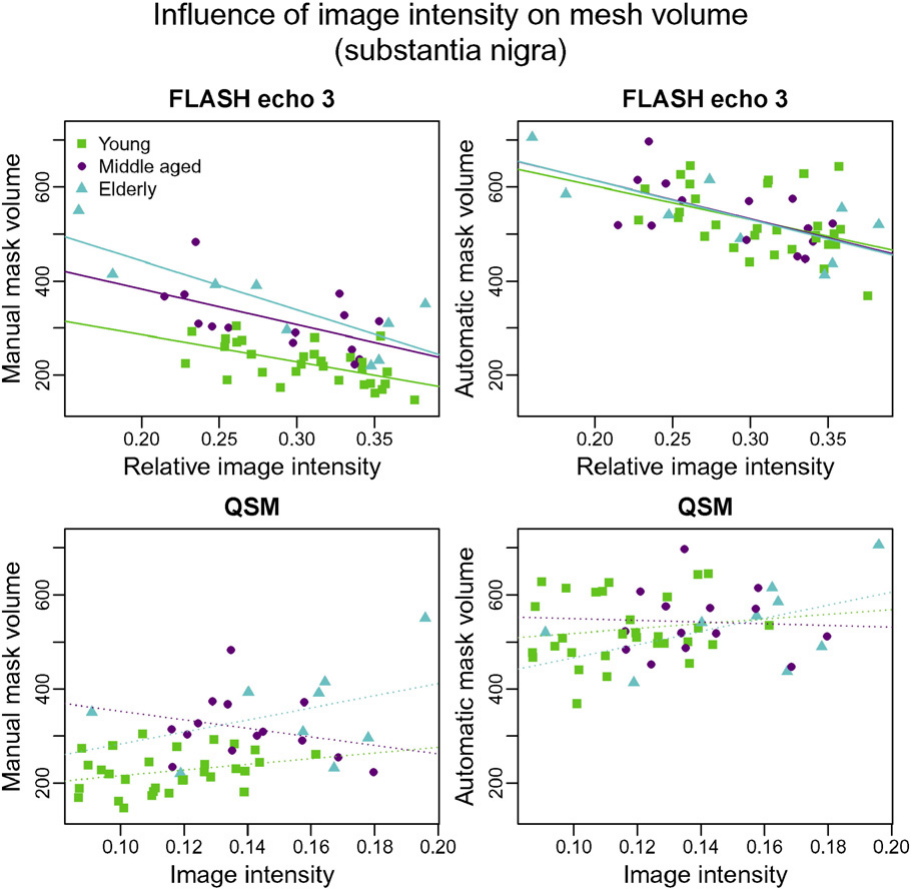
Volumes of manual (column 1) and automatic (column 2) segmentations of the substantia nigra as a function of the image intensity within the structure. Image intensities for the FLASH volumes were normalised through division by the average WM intensity. Only echo 3 is shown as results for echo 1 and 2 are very similar. Green: young, magenta: middle-aged, cyan: elderly. Regression lines are solid if significant (*p* ≤ 0.05) and dotted otherwise.

**Fig. 8:**
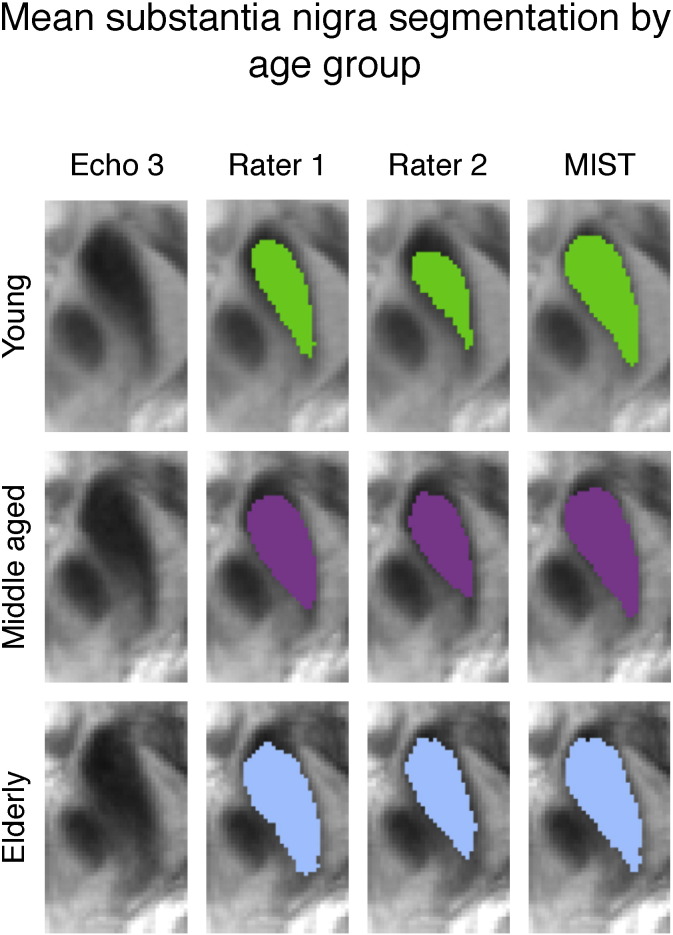
Mean of substantia nigra segmentations for both raters and automatic segmentation, thresholded at 0.5 and overlaid on group mean of FLASH echo 3. Left: young, middle: middle-aged, right: elderly.

The influence of image intensity for the substantia nigra is examined in Fig. 7. There is considerable variation between participants in the image intensity inside the structure in both the *T*_2_^⁎^-weighted and QSM images. Both the volumes of manual and automatic segmentations correlate with the intensity differences, which indicates that both types of segmentation have a degree of sensitivity to image intensity. The separation between the regression lines for the manual segmentations suggests that there may be volume differences between the age groups that are not explained by intensity. This is further illustrated by Fig. 8. Image contrast is fainter in the young group than in the older groups. Despite the intensity differences, MIST includes a more anterior region than the manual segmentations in all age groups. Visual inspection of the surfaces implies that it is not unlikely that this anterior part is part of the substantia nigra.

**Fig. 9:**
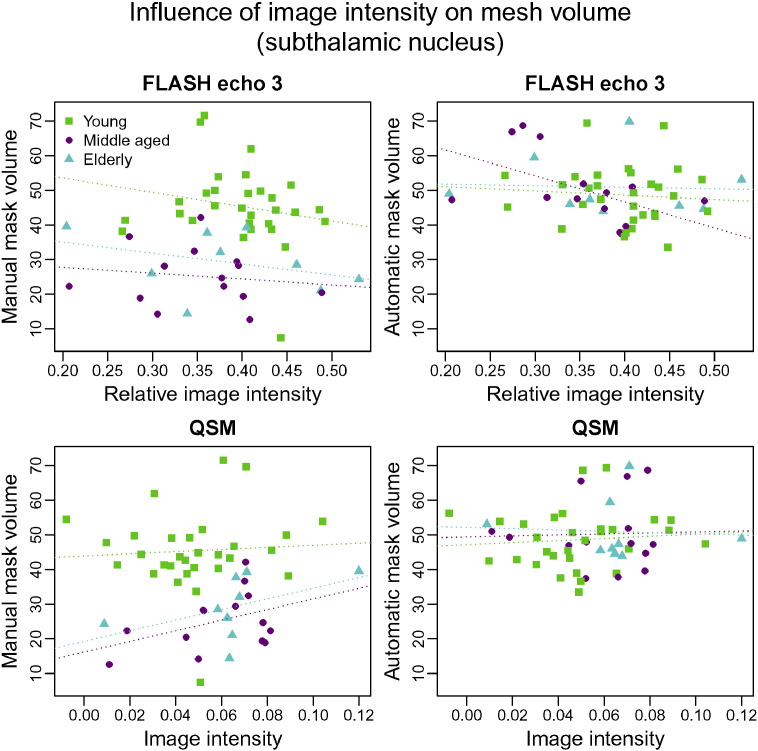
Volumes of manual (column 1) and automatic (column 2) segmentations of the subthalamic nucleus as a function of the image intensity within the structure. Image intensities for the FLASH volumes were normalised through division by the average WM intensity. Only echo 3 is shown as results for echo 1 and 2 are very similar. Green: young, magenta: middle-aged, cyan: elderly. Regression lines are solid if significant (*p* ≤ 0.05) and dotted otherwise.

**Fig. 10:**
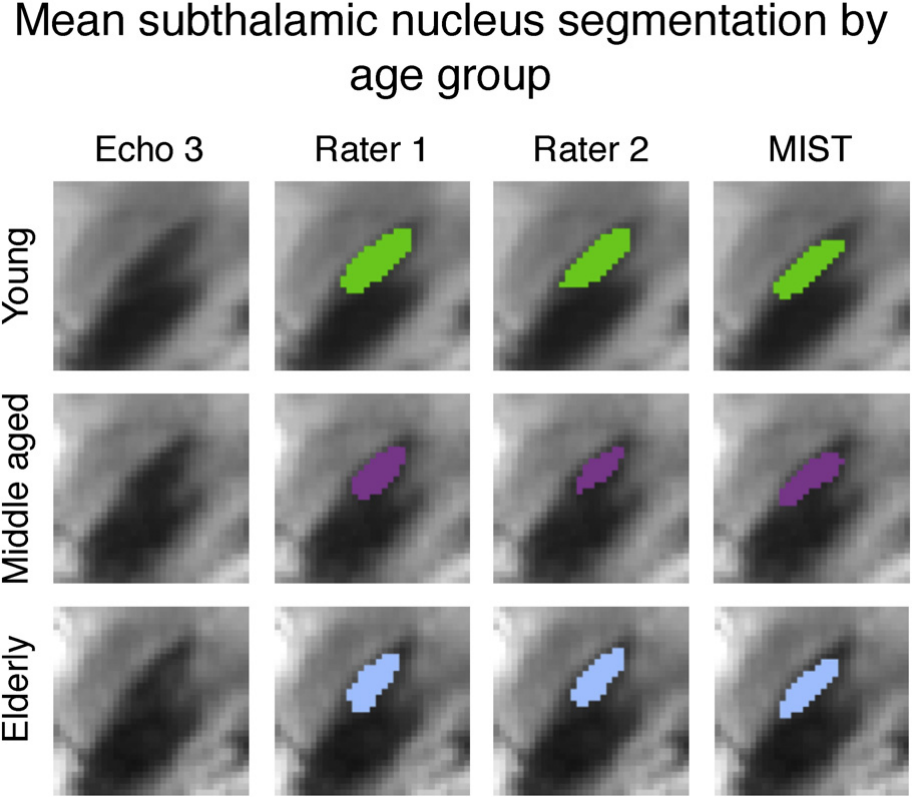
Mean of subthalamic nucleus segmentations for both raters and automatic segmentation, thresholded at 0.5 and overlaid on group mean of FLASH echo 3. Left: young, middle: middle-aged, right: elderly.

For the subthalamic nucleus, there are no significant correlations between image intensity and segmentation volume (Fig. 9). This is likely to be a result of the large degree of variability in both the manual and automatic segmentations that is not related to anatomy (see Section 3.2). The manual segmentations of the subthalamic nucleus appear to be larger in the young group than in the other two groups. An earlier analysis by Keuken et al. (2013) has shown that this difference does not persist when controlling for *T*_2_^⁎^ differences and it appears that automatic segmentation is more consistent across groups. This is further illustrated by Fig. 10.

**Fig. 11:**
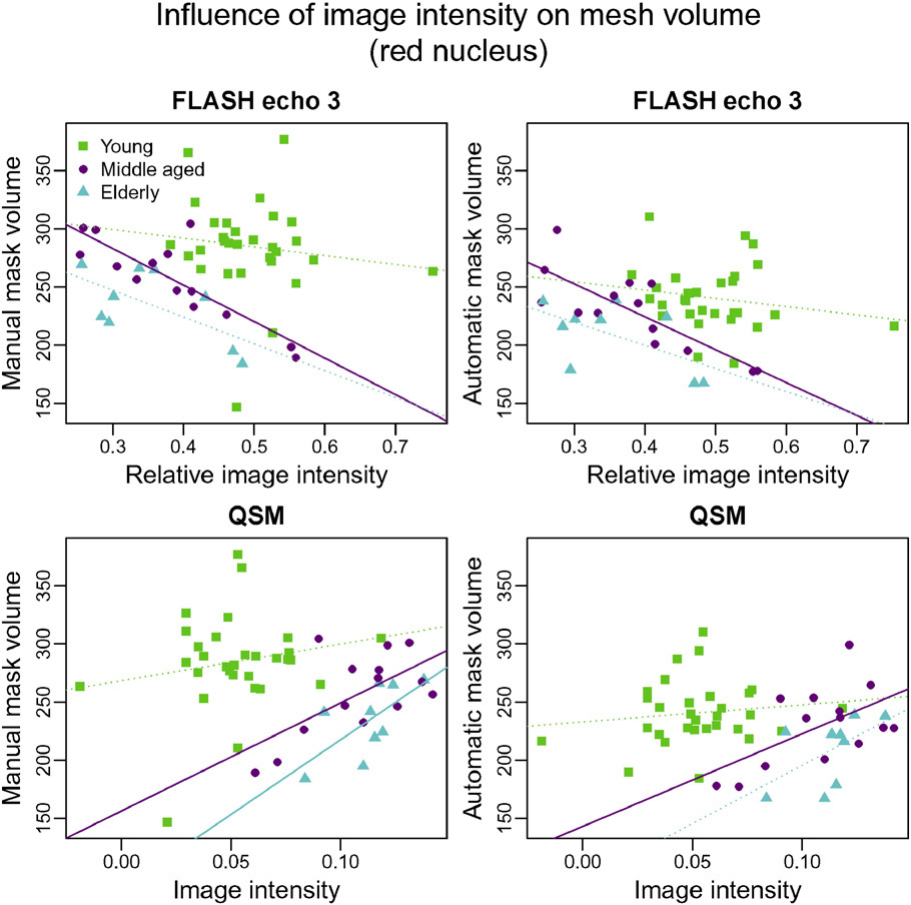
Volumes of manual (column 1) and automatic (column 2) segmentations of the red nucleus as a function of the image intensity within the structure. Image intensities for the FLASH volumes were normalised through division by the average WM intensity. Only echo 3 is shown as results for echo 1 and 2 are very similar. Green: young, magenta: middle-aged, cyan: elderly. Regression lines are solid if significant (*p* ≤ 0.05) and dotted otherwise.

In the red nucleus, the effect of intensity differences is very similar for manual and automatic segmentation, confirming the high degree of correspondence observed in the previous comparisons of red nucleus results (Fig. 11).

## Discussion

We have shown that MIST can successfully segment the substantia nigra, subthalamic nucleus and red nucleus. The dataset we have used is state-of-the-art, but with the increased adoption of 7 T MR imaging systems for research, we expect similar acquisition protocols to become much more widely available in the near future. Because MIST is a multimodal method, it is straightforward to combine the images with different echo times as well as the QSM reconstruction to produce high-quality segmentations. The automatic parameter setup that was described in this paper can be used to automatically retrain the method for other datasets that have sufficient resolution and that include *T*_2_- or *T*_2_^⁎^-weighted images and/or QSM.

### Segmentation quality

High Dice scores were obtained by MIST for the red nucleus, which is clearly visible on both the FLASH magnitude images and the QSM reconstruction. There is little ambiguity in determining what the proper boundaries of the structure should be, as it has a clearly defined globular shape and a sharp boundary on all sides.

Good scores were also obtained for the substantia nigra. These were somewhat lower than those for the red nucleus, which is likely to be due to the fact that its boundaries are less clear than those of the red nucleus in the *T*_2_^⁎^-weighted volumes. There is also considerable intensity variation between different parts of the structure.

Automatic and manual segmentation also exhibit a high degree of overlap for the subthalamic nucleus, although the scores are lower than for the other two structures. The structure is very small and in many cases its exact extent is difficult to see in an image due to lack of clearly defined boundaries in the images. In the example in Fig. 3, it seems that the manual segmentations are slightly more inclusive than those produced by MIST.

The differences in Dice scores between structures can partly be explained by the fact that larger structures (in terms of number of voxels) will in general produce higher scores for comparable errors. This is likely to be a contributing factor to the lower scores for the subthalamic nucleus compared to the red nucleus, for which Dice scores are high. In the case of the substantia nigra, the geometry of the structure also appears to be an important factor. The less compact and more irregular shape of the structure means that errors at the boundary will have a stronger influence on the Dice score.

To investigate how successful automatic segmentation is in capturing anatomical variability, we compared the volumes of the automatic segmentations to those of the manually created masks. We have previously shown how this approach can supplement Dice scores (Visser et al., 2016). Research questions often focus on identifying anatomical correlates and predictors of conditions such as disease or cognitive traits. For the method to be suitable for such studies, the degree to which variability is captured is an important property of the method. The results presented in the present paper show that MIST describes the anatomical variability well for both the substantia nigra and the red nucleus.

### Shape analysis

The mesh-based comparison of the automatic red nucleus segmentations between young and older participants indicates that the volume difference between the groups is not the same along all anatomical axes. The size of the red nucleus decreases primarily along the inferior-superior and anterior-posterior axes with age (Fig. 6). Given that the overall volume of the structure also appears to decrease with age (Fig. 11), this indicates that atrophy occurs in the areas identified in the shape analysis.

The increase in thickness of the substantia nigra is not accompanied by an increase in overall volume in the automatic segmentations (Fig. 6, Fig. 7). This appears to indicate that the structure loses some of its sheet-like characteristic with age and instead becomes slightly thicker and rounder. It is not possible to determine from this pattern whether this change occurs specifically in either one of the major subregions of the substantia nigra, the pars compacta and pars reticulata.

### Influence of image intensity

Image intensity in both the red nucleus and substantia nigra changes considerably with age. The increase in susceptibility and decrease in intensity in the *T*_2_^⁎^ contrast are consistent with an increase in iron content at an older age. This is in agreement with Aquino et al. (2009); Hallgren and Sourander (1958); Pfefferbaum et al. (2009); Schenker et al. (1993); Zecca et al. (2004); Xu et al. (2008), although the changes in image intensity appear to level off at older age in the dataset that we used. Differences may be present in the subthalamic nucleus as well, although the effect is weaker in this structure.

Given that image intensity changes with age, the question arises what influence this change has on both automatic and manual segmentations. The scatter plots in Fig. 7, Fig. 9, Fig. 11 indicate that there is a relationship between image intensity and volume for both the automatic and manual segmentations. The effect of image intensity within each age group is similar for manual and automatic segmentation. Unlike the automatic segmentations however, the manual segmentations of the substantia nigra show a difference in volume between age groups. Fig. 8 suggests that the difference in the appearance of images from the different age groups may have resulted in the manual raters making subtly different labelling decisions for the young participants compared to the older ones.

The phase-based QSM volumes offer a useful image contrast in addition to the magnitude components of the FLASH acquisitions. QSM has been shown to have good reproducibility in the basal ganglia and good correspondence to post mortem measurements of iron concentration (Santin et al., 2016, Langkammer et al., 2012). A second advantage of QSM is that it can reduce the blooming effect induced by variations in tissue susceptibility though deconvolution with a dipole (Li et al., 2012). This will result in sharper edges in the image and our method can learn about these automatically. The sharper profiles measured on the QSM images will result in a narrower probability distribution on the displacements *δ*_*i*_ in the intensity model, which will effectively increase the weight of the QSM data in the segmentations. The sharpness of the QSM images may differ to some degree depending on the reconstruction algorithm that is used (see De Rochefort et al. (2010) for an earlier alternative to Schweser et al. (2013)). The resulting differences in edge appearance are learnt automatically by the method in the training stage.

### General considerations

In this paper we introduced an updated version of MIST that replaces the original shape model as described in Visser et al. (2016) by an MRF-based model that offers more direct control over the smoothness of the segmentations that are produced. The model is no longer regularised by the displacements that were seen in the training data. Instead, the only constraints that are used when segmenting a new image are those imposed by the smoothness of the MRF. There appears to be enough information in the images to produce reliable segmentations without taking shape variations in the training data into account and by removing this constraint, the method should better able to capture unique variations in shape.

The high resolution of the dataset used in this paper is advantageous given that our structures of interest are very small. In particular, while it may be possible to locate the subthalamic nucleus on lower-resolution data, accurate delineation will likely not be as successful. The substantia nigra and red nucleus are slightly larger structures and their segmentation may not require the same high resolutions, although high-quality data will still be important.

In addition to differences in image intensity, the signal-to-noise ratio (SNR) in the images is also likely to affect segmentation performance. Within a study, the same acquisition parameters and hardware are typically used for all participants, however, and this means that the effect of image noise will be comparable for all participants. Any small differences that may exist are not likely to be correlated with biological factors and will not cause problems when interpreting differences in the sizes and shapes of structures. This is unlike the differences in intensity that do exist between participants and which may have biological causes.

Xiao et al. (2012), Haegelen et al. (2013), Xiao et al. (2014) and Kim et al. (2014) describe different methods for automatic segmentation of the substantia nigra, subthalamic nucleus and red nucleus. Although all of these studies use *T*_2_- or *T*_2_^⁎^-weighted MRI data, the imaging resolution and the MR contrasts that are used vary between studies. The Dice score is most commonly reported, but comparison of such scores between studies is problematic due to differences in field strength and acquisition parameters, as well as in the manual segmentations that are used. Despite these limitations, the scores may help in interpreting the results presented in this paper and the scores in Fig. 2, Fig. 3, Fig. 4 are of comparable size to the mean Dice scores reported in these studies, which were in the ranges of 0.57–0.81 for the substantia nigra, 0.58–0.77 for the subthalamic nucleus and 0.78–0.90 for the red nucleus. It should be noted that all of these studies used fairly small samples for evaluation (10 subjects or less) and that most participants were older PD patients. The difficulty in comparing Dice scores between studies is illustrated by the fact that some of the reported scores are higher than the overlap between the two manual raters in the present study. This is likely to be a result of technical differences between the datasets or different manual segmentation procedures. We will release the enhanced version of MIST to the scientific community and this will allow future papers to compare other methods to MIST using the same data for both.

### Conclusion

The results shown in this paper reveal that MIST can produce high quality segmentations of the substantia nigra, subthalamic nucleus and red nucleus in modern 7 T data. In addition, they suggest that the automatic segmentations are at least as accurate as manual delineations and may be less sensitive to confounding differences in image intensity between participants. The enhanced version of MIST will be included in an upcoming release of FSL (http://fsl.fmrib.ox.ac.uk/).

## Figures and Tables

**Table 1:** Age and gender of participants per group. SD denotes standard deviation.

Group	Participants	Male/female	Age in years (SD)
Young	30	16/14	23.8 (2.3)
Middle-aged	14	7/7	52.5 (6.6)
Elderly	9	6/3	69.2 (4.7)

**Table 2:** Rules used to set up the priors for the intensity model. Self refers to the modal intensity inside the structure that is to be segmented. The image intensities for the QSM volumes are not image-based because of the quantitative nature of the modality. No units are shown as the values have not been scaled to physical units and are treated as arbitrary units instead.

Structure	Modality	Type	Inside	Outside
Substantia nigra	*T*_2_^⁎^	Exponential (1 and 3 mm)	Self	Self × 1.33
Exponential (1 mm)	Self	Self × 0.67
QSM	Exponential (1 and 2 mm)	0.15	0.05
Subthalamic nucleus	*T*_2_^⁎^	Exponential (1 and 3 mm)	Self	Self × 1.2
Exponential (1 mm)	Self	Self × 0.5
QSM	Step	0.1	0.0
Exponential (1 and 3 mm)	0.1	0.0
Red nucleus	*T*_2_^⁎^	Exponential (1 and 3 mm)	Self	Self × 1.33
QSM	Exponential (1 and 3 mm)	0.1	0.0

**Table 3:** Additional model parameters. See Visser et al. (2016) for the definitions of the symbols.

Part of model	Parameter	Symbol	Value
Both	Step size	ℓ	Half of voxel size
Number of steps	Δ	$2\times \left\lfloor 2\;\mathrm{mm}/\ell \right\rfloor$
Shape model	MRF weight (substantia nigra and red nucleus)	*w*	10
MRF weight (subthalamic nucleus)	*w*	100
Intensity model	Prior standard deviation (*T*_2_^⁎^)	$\sqrt{\beta /f}$	0.1
Prior standard deviation (*QSM*)	$\sqrt{\beta /f}$	0.0003
Weight of prior mean	*n*_0_	3
Wishart shape parameter	*α*^0^	$\frac{k-1}{2}+3$
Dirichlet parameter for all components	*α*	2
Width of prior on *δ*	*σ*_*δ*_	2 mm
Smoothness	*σ*^*I*^	0.5
